# Gastrointestinal and Hepatic Manifestations of Primary Immune Deficiency Diseases

**DOI:** 10.4103/1319-3767.61230

**Published:** 2010-04

**Authors:** Saleh Z. Al-Muhsen

**Affiliations:** Prince Naif Center for Immunology Research, Department of Pediatrics, College of Medicine, King Saud University, Riyadh, Saudi Arabia

**Keywords:** Chronic granulomatous disease, colitis, common variable immunodeficiency, gastrointestinal manifestation, hepatic involvement, primary immune deficiency diseases, Saudi Arabia

## Abstract

Primary immune deficiency diseases (PIDs) are a heterogeneous group of inherited diseases characterized by variable genetic immune defects, conferring susceptibility to recurrent infections. They have a vast array of manifestations some of which involve the gastrointestinal and hepatobiliary systems. These complications can be the consequence of five different factors, namely, infection, autoimmune process, unregulated inflammation, malignancies and complications of therapeutic intervention. They may precede the PID diagnosis and, once developed, they pose high risk of morbidity. Untrained clinicians may treat these manifestations only at the level of their presentation, leaving the PIDs dangerously undiagnosed. In fact, early diagnosis of PIDs and accompanied gastrointestinal and hepatic complications clearly require appropriate treatment, and in-turn lead to an improved quality of life for the patient. To improve the awareness of gastroenterologists and related health care providers about these diseases, we have reviewed herein the complications of different PIDs focusing on gastrointestinal and hepatic manifestation.

Primary immune deficiency diseases (PIDs) are a heterogeneous group of inherited disorders with unique genetic defects in the immune system. They confer susceptibility to recurrent infections with virulent and non- virulent organisms. In addition to infections, they have a wide spectrum of clinical manifestations including autoimmune diseases, unregulated inflammation and predisposition to malignancies.[1–4] PIDs are generally considered uncommon diseases; however, recent data from the Middle East, including Saudi Arabia, indicated that PIDs are 10-20 times more common than worldwide reported figures,[5–8] probably secondary to high incidence of consanguinity, reaching up to 50% in some countries.[9–13]

Currently, more than 140 distinct genes have been identified, the abnormalities of which account for more than 200 different clinical phenotypes of PID.[2] Since this field is continuously revolutionized with unforeseen discoveries of novel PIDs and the characterization of their molecular defects, The International Union of Immunological Societies (IUIS) has recently updated the classification of PIDs [Table 1]

**Table 1:** Gastrointestinal and hepatic manifestations of primary immune deficiency diseases, based on the most recent classification of PIDs

**Category of PID**§	**Primary immunodeficiency disease**	**GI manifestation**
Combined T and B cells immunodeficiencies	Severe combined immunodeficiency Omenn syndrome ADA deficiency MHC-II deficiency (Bare lymphocyte syndrome) Hyper IgM syndrome	Colitis and hepatitis (CMV), candidiasis, chronic diarrhea, GvHDDiarrhea, hepatosplenomegaly, esinophilic enteropathyHepatitis (autoimmune, toxic), diarrheaProtracted diarrhea (*Cryptosporidium*), progressive liver disease, sclerosing cholangitisOral ulcers, diarrhea (*Cryptosporidium*), progressive liver disease, sclerosing cholangitis
Predominantly antibody deficiencies	Selective IgA deficiencyX-Linked agammagloulinemiaCVID	Diarrhea (*Giardia*), celiac sprue, nodular lymphoid hyperplasiaGI diseases are chronic diarrhea and malabsorptionDiarrhea (*Giardia*), nodular lymphoid hyperplasia, villous blunting, IBD-like colitis, pernicious anemia
Other well-defined immunodeficiencies	Wiskott–Aldrich syndromeHyper-IgE syndromeChronic mucocutaneous candidiasisHepatic veno-occlusive disease with immunodeficiency	Colitis, bloody diarrhea, esinophilic enteropathy, lymphomaPeriodontitis, liver abscesses, esinophilic enteropathyEsophageal candidiasisHepatic veno-occlusive disease, hepatosplenomegaly
Diseases of immune dysregulation	IPEXHemansky–Pudlak syndromeXLP	Severe enteropathy, diarrhea, malabsorption and failure to thriveGranulomatous colitisPost-EBV fulminant hepatic failure, hepatosplenomegaly, lymphoma
Disorders of phagocytes	Chronic granulomatous disease	Oral ulcers, esophageal dysmotility, gastric outlet obstruction, small bowel obstruction, colitis, perianal fistula and abscesses, hepatitis, liver abscesses
	Leukocyte adhesion defectShwachman–Diamond syndrome	Omphalitis, periodontitis, perianal ulcersPancreatic enzyme insufficiency, diarrhea, malabsorption
Defects in innate immunity	IFN-γ and IL-12 circuit defect (MSMD)	Salmonella gastroenteritis, mycobacterial liver and spleen abscesses
Autoimmunity disorders	Periodic fever syndromeBlau syndrome	Peritonitis, abdominal painCrohn's disease
Complement deficiencies	Hereditary angioedema	Intestinal wall edema, severe abdominal pain

§ The complete, updated IUIS PID classification.[2]

*Abbreviations used: CMV= Cytomegalovirus; GvHD= Graft versus host disease; ADA= Adenosine deaminase deficiency; MHC-II- Major histocompatibility complex-II; CVID= Common variable immunodeficiency; IBD= Inflammatory bowel disease; IPEX= Immunodeficiency, Polyendocrinopathy, Enteropathy, X-linked; XLP= X-Linked lymphoproliferative syndrome; EBV= Epstein Barr virus; MSMD= Mendelian susceptibility to mycobacterial disease.

It is important for clinicians to be aware of the various manifestations of PIDs. Early recognition and diagnosis is vital in improving the quality of life and wellbeing of these patients.

## GENERAL CONCEPTS

The most common manifestation of PIDs is usually respiratory in nature; however, with advances in diagnostic tools, appropriate antimicrobial therapy and intravenous immunoglobulin replacement therapy, respiratory infections have been fairly controlled. The next system commonly affected in PIDs is the gastrointestinal (GI) system, serving as a primary barrier to infections and considered the largest immune organ of the body.[14]

The GI complications of PIDs can present in five different forms: (1) infection throughout the GI tract or hepatobiliary system such as giardiasis in humoral immune dysfunction; cytomegalovirus colitis and hepatitis in severe T cell dysfunction as well as hepatic abscess in phagocytic defect. (2) Autoimmune phenomena as seen in autoimmune hepatitis and enteropathy associated with some PIDs. (3) Unregulated inflammatory conditions such as granulomatous colitis in CGD. (4) Malignancies involving the GI tract and hepatobiliary system.(5) GI and hepatic complications secondary to therapeutic intervention, for example, liver or gut graft-versus-host-disease and veno-occlusive disease post hematopoietic stem cell transplantation in certain PIDs. Nevertheless, addressing the variable manifestations of PIDs is beyond the scope of this review. We will therefore focus on a few examples of PID diseases commonly present with GI and hepatic manifestation, which might be the initial presentation of the disease rather than as part of the constellation of symptoms PIDs are known for.[15] Further examples of PIDs presenting with GI and hepatic manifestation are summarized in Table 1.

## CHRONIC GRANULOMATOUS DISEASE

Chronic granulomatous disease (CGD) is a primary immunodeficiency caused by a genetic defect in one of the components of NADPH oxidase of the phagocytic cells. This important complex is responsible for the generation of superoxide and is involved in combating catalase producing organisms such as many bacteria and fungi.[16–19] Five genetic mutations involving the phagocytic oxidase system have been identified so far. The most common is an X-linked recessive defect in gP91phox, while three other autosomal recessive (AR) defects were reported in P22phox, P47phox and P67phox components of the NADPH oxidase system.[20–25] A novel mutation in *NCF4*, the gene encoding P40phox, has also recently been reported in a boy who presented with granulomatous colitis, delineating the fourth AR form of CGD.[26] In addition to susceptibility to infections, CGD patients are prone to develop noninfectious complications characterized by unregulated inflammation such as granulomatous colitis, chorioretinal lesions and lupus-like disease.[27–29]

### Gastrointestinal manifestations in CGD

GI manifestation is commonly encountered among CGD patients and might even precede the CGD diagnosis.[30] It usually arises from an abnormal inflammatory response leading to exuberant granuloma formation. In a study of 140 patients, GI manifestation was recorded in 46 patients (32.8%).[30] Abdominal pain, vomiting, diarrhea and weight loss, although nonspecific, were common GI symptoms among these CGD patients.[30–32] Moreover, we have observed that 8 (15%) of 55 CGD patients had colitis and/or GI obstruction (unpublished data).[33]

#### Proximal gastrointestinal tract manifestations

Granulomatous stomatitis, oral ulcers and dental abscesses are often found in these children.[30,32] Oral candidiasis has also been documented.[34] Affected individuals may present with granulomatous inflammation causing obstruction and stricture formation throughout the entire GI tract. Therefore, it is not unusual for these patients to present with obstructive upper GI symptoms such as dysphagia, dysmotility, delayed emptying and vomiting.[31–32] Other gastric complications include eosinophilic gastritis and abscesses have been rarely reported.[35]

#### Intestine and colon manifestations

**Figure 1:**
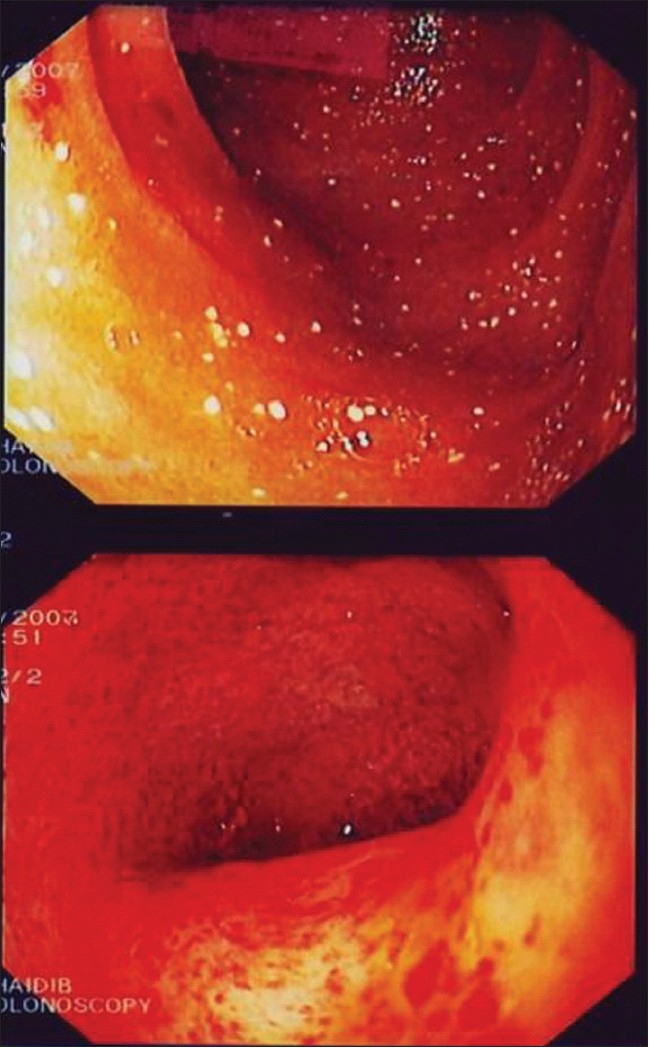
Gastrointestinal abnormalities in CGD. Colonoscopic finding of mucosal inflammation, erythema, edema, ulceration and loss of normal vascular pattern

**Figure 2:**
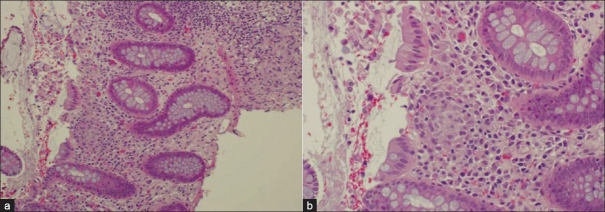
(a) Histopathological findings revealing chronic inflammation, architectural distortion (b) High power field showing small non-necrotizing granuloma

Granulomatous colitis is prevalent among CGD patients, especially X-linked variant CGD.[29] The endoscopic finding is similar to inflammatory bowel disease (IBD), particularly, Crohn's disease (CD) characterized by transmural patchy inflammatory “skip lesions” with intact segments between the diseased ones [Figure 1]. These inflammatory lesions might cause obstruction, stenosis and fistula.[36] The difference between CD and CGD colitis lies in the histopathology of the granuloma formation. CGD colitis is characterized by sharply defined aggregates of epithelioid histiocytes surrounded by a cuff of dense lymphocytic inflammation [Figure 2]. In CD, granulomata are poorly defined.[30] One group claims that the main difference lies in the presence of pigment-laden macrophages within the lamina propria.[36] Of note, the inflammatory infiltrates of this form of colitis were mainly eosinophils and macrophages. There is also an increased expression of HLA-DR in the epithelium and vascular endothelium.[37] Nevertheless, the presentation is very similar to that of IBD, more specifically, CD, to an extent that it fulfills the Lennard–Jones criteria for CD. Furthermore, a subset of CGD patients may have IBD symptoms as their initial presentation.[30,32,34]

#### Distal gastrointestinal tract manifestations

Fistulae–in–ano is common probably due to the high bacterial load in the perianal area resulting from ineffective clearance of bacteria in CGD patients. It usually develops following infection of the perianal glands or granulomatous inflammation arising from the rectum. Steroids and interferon-γ have been shown to be effective in controlling the GI granulomatous inflammation among CGD patients, and may reduce the thickening and relieve the obstruction.[30,32] However, large scale prospective studies are required to validate their safety and efficacy.

#### Growth delay

Another consequence of chronic GI inflammation among children with CGD is growth delay.[30] In a large CGD cohort with GI complication, 32% had at least 1 height measurement below the fifth percentile and 22% had at least 1 weight measurement below the fifth percentile during their study period. Furthermore, CGD patients with GI involvement are more prone to suffer growth delay compared to those who are unaffected.[30]

### Hepatobiliary manifestations in CGD

Transient elevation of liver enzymes is a common incidental finding in CGD.[38] One of the contributing factors is recurrent liver abscesses, which are frequently encountered among CGD patients.[29] Liver abscesses were reported in 25% to 45% of CGD patients, and were correlated with high rate of mortality reaching up to 27% in some studies despite appropriate antimicrobial therapies.[29,31] According to the national US registry, *Staphylococcus aureus*, *Pseudomonas aeruginosa, and Burkholderia cepacia* were the most common pathogens isolated from these abscesses. Other infections with *Serratia, Aspergillus, Candida and Mycobacteria* were also encountered.[29,38–39] Typically, these abscesses relapse frequently, but at a new location in the liver.[38] They are dense, fibrotic, caseous, difficult to drain and almost always require surgery.

The elevation in liver enzymes cannot be attributed only to infections. Hussain *et al,* reported 29 CGD patients who developed drug-related hepatotoxicity.[38] Further studies are needed to explore the substantial risk of drug hepatotoxicity in CGD patients, as a majority of CGD patients will need long-term antibiotic and antifungal prophylactic therapies.[39–42]

In summary, CGD has variable GI manifestations. In addition to infections, exuberant granulomatous inflammation may occur anywhere along the GI tract, which is very similar to CD in presentation. In certain circumstances this might be complicated with obstruction. These patients will be burdened with abdominal pain, nausea, vomiting, diarrhea and constipation. Eventually, GI complications lead to growth delay and failure to thrive. Chronic liver abscess is a significant risk for morbidity in CGD that warrants long-term antibiotic therapy and surgical intervention in some instances. Another complication that should be dealt with in these patients is liver injury due to drug-induced hepatitis.

CGD was formerly associated with high mortality but current practice of antimicrobial, IFN-γ prophylaxis, aggressive surgery and early hematopoietic stem cell transplantation or gene therapy have improved the outcome substantially.[39–47]

## COMMON VARIABLE IMMUNODEFICIENCY

Common variable immunodeficiency (CVID) is the second most prevalent PID. It is characterized by dysfunction of the humoral immunity with low B lymphocytes, hypogammaglobulinemia and/or impaired antibody response to infection and vaccination. Affected individuals are therefore vulnerable to recurrent respiratory infections, chronic diarrhea and autoimmune manifestations. In fact, the immune defect is not limited to immunity cells; there is a clear crucial role for T cell defect in CVID as well. Moreover, most GI manifestation is probably related to T cell dysfunction explaining the poor response to intravenous immunoglobulin (IVIG) alone.[48–49] Despite its prevalence and the plethora of literature on CVID, it is not fully elucidated at the molecular level. The underlying genetic defects have been explored in only a small subset of CVID. Mutations in three genes, *TACI* (transmembrane activator and calcium-modulator [CAML] and cyclophilin ligand Interactor),[50] *ICOS* (an inducible costimulator on activated T cells)[51] and CD19 genes, were found to cause only 10% of CVID cases.[52]

### Gastrointestinal manifestations

Several studies showed that the prevalence of GI manifestation in CVID is quiet significant, ranging from 20% to 50%.[48,53–54] Noteworthy to mention is that these GI complications pose high morbidity and come second only to respiratory complications.

#### Proximal gastrointestinal tract manifestations

Although the oral cavity and esophagus are not commonly affected in CVID, the prolonged antibiotic administration, with accompanying neutropenia, predispose to fungal infections, especially esophageal candidiasis.[49,55]

Atrophic gastritis and achlorhydria are common ailments, reported in about 50% of the cases among CVID patients.[48,53] Pathologic studies revealed mild to moderate infiltration of lymphomononuclear cells in the lamina propria, lack of plasma cells and increased apoptosis.[48–49,55] Reduced serum gastrin levels and pernicious anemia-like syndrome might develop eventually.[48,53] CVID disease significantly increases the risk for gastric carcinoma that might reach up to 50-fold. Concomitant *Helicobacter pylori* infection increases this risk substantially.[48,56]

#### Small intestine manifestations

Chronic diarrhea is the most common GI manifestation, and may be the solo presentation of CVID. Several factors contribute to chronic diarrhea, including: Celiac sprue- like disorder, *Giardia* infection, bacterial overgrowth and, rarely, small bowel lymphoma.[48] Histopathologic studies consistently report villous blunting; however, there is absence of plasma cell infiltration.

*Giardia lamblia* is a prominent pathogen in the small bowel disease among CVID patients, resulting in abdominal cramps, bloating and watery diarrhea.[48] Despite empiric therapy with metronidazole, diarrhea is minimally resolved due to high recurrence of infection.

Autoimmune enteropathy is another distinct CVID manifestation with auto-antibodies directed against the enterocyte, thus further contributing to chronic diarrhea.[48–49] Moreover, nodular lymphoid hyperplasia (NLH) resulting from failure of B cell follicle formation, occurs commonly in CVID. It manifests as multiple polyps or nodules in the small intestine, but could also be seen in the stomach and colon.[49,56–57]

#### Large intestine manifestations

**Figure 3:**
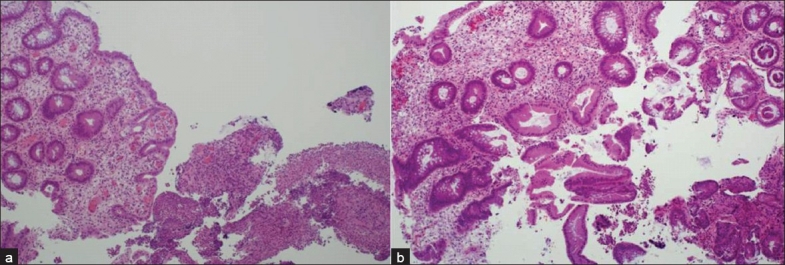
Histopathological features of colitis in common variable immunodeficiency. Colon biopsy showing (a) acutely inflamed colonic tissue and colonic mucosa with chronic colitis. (b) Chronic colonic crypt damage manifested as branching, tangential alignment and increased amount of chronic inflammatory cells and absence of plasma cells and giant granuloma

IBD is frequently encountered in CVID. Colitis due to CVID is distinct from other forms of colitis. Nevertheless, it mimics ulcerative crypt destructive colitis with regard to certain features. On examining histopathological biopsies, one can usually observe an increase in lymphocyte and macrophage inflammatory infiltration as well as overexpression of apoptosis; however, granulomas and giant cells are usually absent [Figure 3a and b].[48,55] The underlying mechanisms may involve T cell defect and autoimmune phenomenon; therefore, IVIG alone does not control the symptoms. Steroids and immunosuppressive therapies are often used with reasonable response.

### Malignancy

CVID has substantial risk of GI malignancies. There is a 50-fold higher risk of gastric adenocarcinoma among CVID patients.[58] Contributing factors include achlorhydria, intestinal metaplasia and pernicious anemia. Early monitoring with endoscopies is therefore essential, especially for patients manifesting with constitutional symptoms such as weight loss or anorexia. Moreover, risk for B cell immunophenotype lymphoma triggered by Epstein-Barr virus is 30 times higher in CVID patients as well.[48,58–59]

## HYPER IgM SYNDROME

Hyper IgM (HIGM) syndrome is a rare primary immunodeficiency disease caused by impaired Immunoglobulin class switch recombination (CSR) and characterized by normal or high IgM along with low or undetectable immunoglobulin subtypes including IgG, IgA and IgE.[60] Different molecular causes have been described that result in different HIGM phenotypes including defects of CD40 ligand (CD40L), CD40, nuclear factor-ĸB essential modulator (NEMO), activation-induced cytidine deaminase and uracil-DNA glycosylase.[61–67] Addressing different genotypes and phenotypes of HIGM is beyond the scope of this review and was well reviewed by Erdos *et al*.[60]

Infections dominate this type of immunodeficiency, which involve the upper and lower respiratory tract, commonly caused by *Pneumocystis jiroveci pneumonia (PJP)*.[68] The other most commonly documented clinical manifestations are chronic diarrhea, liver involvement and neutropenia. Lymphoid hyperplasia is also a noteworthy finding in HIGM.[69]

### Gastrointestinal manifestations

HIGM patients present frequently with oral ulcers, gingivitis and rectal ulcers, which are probably attributed to neutropenia that commonly complicate HIGM. Fifty percent of HIGM patients experience GI problems, mainly protracted diarrhea, mostly due to infections with *Cryptosporidium parvum, Giardia lamblia, Salmonella* and *Entamoeba histolytica.*[68–69] Other noninfectious causes of diarrhea such as IBD and intestinal nodular bowel disease have been reported.[68]

### Liver involvement

Hepatic involvement is a very prominent complication of HIGM. Although sclerosing cholangitis (SC) occurs rarely among children, 10% of these cases are more often associated with primary immunodeficiencies.[70] It is suspected that in sclerosing cholangitis, bile ducts undergo inflammation, which progress to fibrosis leading to cirrhosis and liver failure. *C. parvum* is the most common pathogen involved in SC.[68]

### Malignancy

The exact underlying mechanism for the susceptibility of HIGM patients to biliary tract carcinoma remains elusive. It is hypothesized that long standing cryptosporidium infection may cause epithelium bile duct dysplasia.[71] Hepatitis B, C, and CMV infections were also documented along with autoimmune hepatitis, to possibly progress to hepatocellular carcinoma. SC and malignant tumors of the liver, biliary tree and pancreas are predictors of poor outcome in HIGM.[71]

In conclusion, diarrhea and sclerosing cholangitis are the most prominent findings among children with hyper IgM. Careful monitoring is warranted, especially with cryptosporidium infection, since this pathogen may result in grave consequences.

## IPEX SYNDROME

IPEX comprises **I**mmunodeficiency, **P**olyendocrinopathy, **E**nteropathy, and occurs as an **X**-linked Mendelian trait.[72] It is a unique syndrome caused by mutation in *FOXP3*, a gene encoding putative deoxyribonucleic acid (DNA)-binding protein of the forkhead family, which acts as transcriptional repressor and key modulator of regulatory T cell function.[72–75] Skin manifestations and other autoimmune phenomena are associated with this syndrome.[74–75] This rare immunodeficiency confers predisposition to infections commonly with *Enterococcus* and *Staphylococcus*.[75]

### Gastrointestinal manifestations

The single most common manifestation of IPEX is intractable diarrhea, which was reported in almost all patients.[75] The diarrhea starts very early and may even precede the initiation of feeding of infants, while becoming worse with feeding. Gluten restriction and parenteral nutrition have been found to minimally improve the prognosis.[76] Histopathologic studies of the small intestine reveal severe villous atrophy and mucosal erosion with lymphocytic infiltrates of the submucosa or lamina propria.[77] Failure to thrive usually follows as a result of the enteropathy and malabsorption.[75] Growth retardation has been suspected to start prenatally, which would explain cachexia as a striking feature of this disease.[76] Currently, there is limited information on the disease profile of IPEX and most patients die within the first year of life. Most treatment strategies are directed toward immunosuppression and/or bone marrow transplantation.[78]

## INFLAMMATORY BOWEL DISEASE AS A PRIMARY IMMUNODEFICIENCY

The examples discussed above represent a group of PIDs that may present with GI manifestations. Colitis resembling IBD is an evident example. On the other hand, there is a new concept postulating that IBD, particularly CD, is not considered solely an inflammatory disease but is also a *bonafide* genetic trait, resulting in an immune defect in the macrophage. It may result from impaired recruitment of granulocytes to the GI wall, causing impaired clearance of the microorganism, and hence contributing to granuloma formation.[79–81] Over the last 2 decades, the genetic cause of CD remains elusive despite the identification of up to 32 candidate loci in wide genome association studies, among which there is robust association with *NOD2/CARD15* gene, encoding the cytosolic receptor that recognizes bacterial peptidoglycans, particularly mycobacterial *N*-glycolyl muramyl dipeptide.[82–83] The new hypothesis focuses on impaired macrophage function, caused by a single gene Mendelian defect, rather than polygenic disease. If this is proven to be correct, it will cause a paradigm shift in our understanding of IBD and shall pave the way for major discoveries to delineate genetic causes of CD.[79]

## CONCLUSION

PIDs have a variety of manifestations, some of which involve the GI and hepatobiliary systems. Infectious and noninfectious GI and hepatic complications pose high risk of morbidity in patients with PIDs. Health care professionals specialized in gastroenterology are not usually involved in the presentation and diagnosis of patients with PIDs. Untrained clinicians may treat these only at the level of its presentation, leaving the PIDs dangerously undiagnosed. Early diagnosis of PIDs and accompanied GI and hepatic complications clearly improve the quality of life for affected patients and allow for appropriate treatments. Multicenter large clinical studies are needed to evaluate the nature of GI and hepatic manifestations in these rare PIDs. Likewise, further studies are needed to evaluate the preventive and therapeutic modalities of PID-associated GI manifestations.

## ABBREVIATIONS

PIDs, primary immune deficiency diseases; GI, gastrointestinal; CMV, cytomegalovirus; GvHD, graft versus host disease; CGD, chronic granulomatous disease; AR, autosomal recessive; CD, Crohn's disease; CVID, common variable immunodeficiency; IVIG, intravenous immunoglobulin; HIGM, hyper IgM; IPEX, immunodeficiency, polyendocrinopathy, enteropathy, x-linked; IBD, inflammatory bowel disease.

## References

[1] Woroniecka M, Ballow M (2000). Office evaluation of children with recurrent infection. Pediatr Clin North Am.

[2] Geha RS, Notarangelo LD, Casanova JL, Chapel H, Conley ME, Fischer A (2007). Primary immunodeficiency diseases: An update from the International Union of Immunological Societies Primary Immunodeficiency Diseases Classification Committee. J Allergy Clin Immunol.

[3] Bonilla FA, Geha RS (2003). 12. Primary immunodeficiency diseases. J Allergy Clin Immunol.

[4] Yarmohammadi H, Estrella L, Doucette J, Cunningham-Rundles C (2006). Recognizing primary immune deficiency in clinical practice. Clin Vaccine Immunol.

[5] Rezaei N, Aghamohammadi A, Moin M, Pourpak Z, Movahedi M, Gharagozlou M (2006). Frequency and clinical manifestations of patients with primary immunodeficiency disorders in Iran: Update from the Iranian Primary Immunodeficiency Registry. J Clin Immunol.

[6] Al-Herz W (2008). Primary immunodeficiency disorders in Kuwait: First report from Kuwait National Primary Immunodeficiency Registry (2004–2006). J Clin Immunol.

[7] Suliaman FA, Harfi H (2006). High incidence of severe combined immune deficiency in the Eastern Province of Saudi Arabia. Pediatr Asthma Allergy Immunol.

[8] Suliaman FA, Sheikh S, Almuhsen S, Alsmadi O (2009). Epidemiology of chronic granulomatous disease of childhood in eastern province, Saudi Arabia. Pediatr Asthma Allergy Immunol.

[9] El Mouzan MI, Al Salloum AA, Al Herbish AS, Qurachi MM, Al Omar AA (2008). Consanguinity and major genetic disorders in Saudi children: A community-based cross-sectional study. Ann Saudi Med.

[10] Koc I (2008). Prevalence and sociodemographic correlates of consanguineous marriages in Turkey. J Biosoc Sci.

[11] Rezaei N, Pourpak Z, Aghamohammadi A, Farhoudi A, Movahedi M, Gharagozlou M (2006). Consanguinity in primary immunodeficiency disorders: The report from Iranian Primary Immunodeficiency Registry. Am J Reprod Immunol.

[12] Hamamy HA, Masri AT, Al-Hadidy AM, Ajlouni KM (2007). Consanguinity and genetic disorders: Profile from Jordan. Saudi Med J.

[13] Kanaan ZM, Mahfouz R, Tamim H (2008). The prevalence of consanguineous marriages in an underserved area in Lebanon and its association with congenital anomalies. Genet Test.

[14] McCabe R P (2002). Gastr ointestinal manifestations of non-AIDS immunodeficiency. Curr Treat Options Gastroenterol.

[15] Agarwal S, Mayer L (2009). Gastrointestinal manifestations in primary immune disorders. Inflamm Bowel Dis.

[16] Bridges RA, Berendes H, Good RA (1959). A fatal granulomatous disease of childhood: The clinical, pathological, and laboratory features of a new syndrome. AMA J Dis Child.

[17] Ochs HD, Edvard SC, Puck JM (1999). Primary immunodeficiency diseases: A molecular and genetic approach.

[18] Landing BH, Shirkey HS (1957). A syndrome of recurrent infection and infiltration of viscera by pigmented lipid histiocytes. Pediatrics.

[19] Johnston RB (2001). Clinical aspects of chronic granulomatous disease. Curr Opin Hematol.

[20] Forehand JR, Curnutte JT, Johnston RB, Scriver CR, Sly WS, Valle D (1995). Inherited disorders of phagocytic killing. The metabolic and molecular bases of inherited disease.

[21] Segal BH, Leto TL, Gallin JI, Malech HL, Holland SM (2000). Genetic, biochemical, and clinical features of chronic granulomatous disease. Medicine (Baltimore).

[22] Baehner RL, Kunkel LM, Monaco AP, Haines JL, Conneally PM, Palmer C (1986). DNA linkage analysis of X chromosome-linked chronic granulomatous disease. Proc Natl Acad Sci U S A.

[23] Roos D, de Boer M, Kuribayashi F, Meischl C, Weening RS, Segal AW (1996). Mutations in the X-linked and autosomal recessive forms of chronic granulomatous disease. Blood.

[24] Cross AR, Noack D, Rae J, Curnutte JT, Heyworth PG (2000). Hematologically important mutations: The autosomal recessive forms of chronic granulomatous disease (first update). Blood Cells Mol Dis.

[25] Heyworth PG, Curnutte JT, Rae J, Noack D, Roos D, van Koppen E (2001). Hematologically important mutations: X-linked chronic granulomatous disease (second update). Blood Cells Mol Dis.

[26] Matute JD, Arias AA, Wright NA, Wrobel I, Waterhouse CC, Li XJ (2009). A new genetic subgroup of chronic granulomatous disease with autosomal recessive mutations in p40phox and selective defects in neutrophil NADPH oxidase activity. Blood.

[27] Curnutte JT (1993). Chronic granulomatous disease: The solving of a clinical riddle at the molecular level. Clin Immunol Immunopathol.

[28] Gallin JI, Buescher ES, Seligmann BE, Nath J, Gaither T, Katz P (1983). NIH conference: Recent advances in chronic granulomatous disease. Ann Intern Med.

[29] Winkelstein JA, Marino MC, Johnston RB, Boyle J, Curnutte J, Gallin JI (2000). Chronic granulomatous disease: Report on a national registry of 368 patients. Medicine (Baltimore).

[30] Marciano BE, Rosenzweig SD, Kleiner DE, Anderson VL, Darnell DN, Anaya-O'Brien S (2004). Gastrointestinal involvement in chronic granulomatous disease. Pediatrics.

[31] Barton LL, Moussa SL, Villar RG, Hulett RL (1998). Gastrointestinal complications of chronic granulomatous disease: Case report and literature review. Clin Pediatr (Phila).

[32] Huang A, Abbasakoor F, Vaizey CJ (2006). Gastrointestinal manifestations of chronic granulomatous disease. Colorectal Dis.

[33] Al-Muhsen SA, Al-Saud B, Al-Ghonaium A, Al-Mousa H, Al-Dhekri H, Al-Gazlan S (2009). Molecular and clinical profile in a large cohort with chronic granulomatous diseases from Saudi Arabia. Keystone symposium on Human Immunology and Immunodeficiencies.

[34] Movahedi M, Aghamohammadi A, Rezaei N, Farhoudi A, Pourpak Z, Moin M (2004). Gastrointestinal manifestations of patients with chronic granulomatous disease. Iran J Allergy Asthma Immunol.

[35] Johnson FE, Humbert JR, Kuzela DC, Todd JK, Lilly JR (1975). Gastric outlet obstruction due to X-linked chronic granulomatous disease. Surgery.

[36] Marks DJ, Miyagi K, Rahman FZ, Novelli M, Bloom SL, Segal AW (2009). Inflammatory bowel disease in CGD reproduces the clinicopathological features of Crohn's disease. Am J Gastroenterol.

[37] Schappi MG, Klein NJ, Lindley KJ, Rampling D, Smith VV, Goldblatt D (2003). The nature of colitis in chronic granulomatous disease. J Pediatr Gastroenterol Nutr.

[38] Hussain N, Feld JJ, Kleiner DE, Hoofnagle JH, Garcia-Eulate R, Ahlawat S (2007). Hepatic abnormalities in patients with chronic granulomatous disease. Hepatology.

[39] Mouy R, Fischer A, Vilmer E, Seger R, Griscelli C (1989). Incidence, severity, and prevention of infections in chronic granulomatous disease. J Pediatr.

[40] Gallin JI, Alling DW, Malech HL, Wesley R, Koziol D, Marciano B (2003). Itraconazole to prevent fungal infections in chronic granulomatous disease. N Engl J Med.

[41] Margolis DM, Melnick DA, Alling DW, Gallin JI (1990). Trimethoprim-sulfamethoxazole prophylaxis in the management of chronic granulomatous disease. J Infect Dis.

[42] Mouy R, Veber F, Blanche S, Donadieu J, Brauner R, Levron JC (1994). Long-term itraconazole prophylaxis against Aspergillus infections in thirty-two patients with chronic granulomatous disease. J Pediatr.

[43] The International Chronic Granulomatous Disease Cooperative Study Group (1991). A controlled trial of interferon gamma to prevent infection in chronic granulomatous disease. N Engl J Med.

[44] Marciano BE, Wesley R, de Carlo ES, Anderson VL, Barnhart LA, Darnell D (2004). Long-term interferon-gamma therapy for patients with chronic granulomatous disease. Clin Infect Dis.

[45] Seger RA, Gungor T, Belohradsky BH, Blanche S, Bordigoni P, Di Bartolomeo P (2002). Treatment of chronic granulomatous disease with myeloablative conditioning and an unmodified hemopoietic allograft: A survey of the European experience, 1985-2000. Blood.

[46] Gungor T, Halter J, Klink A, Junge S, Stumpe KD, Seger R (2005). Successful low toxicity hematopoietic stem cell transplantation for high-risk adult chronic granulomatous disease patients. Transplantation.

[47] Ott MG, Schmidt M, Schwarzwaelder K, Stein S, Siler U, Koehl U (2006). Correction of X-linked chronic granulomatous disease by gene therapy, augmented by insertional activation of MDS1-EVI1, PRDM16 or SETBP1. Nat Med.

[48] Kalha I, Sellin JH (2004). Common variable immunodeficiency and the gastrointestinal tract. Curr Gastroenterol Rep.

[49] Khodadad A, Aghamohammadi A, Parvaneh N, Rezaei N, Mahjoob F, Bashashati M (2007). Gastrointestinal manifestations in patients with common variable immunodeficiency. Dig Dis Sci.

[50] Castigli E, Wilson SA, Garibyan L, Rachid R, Bonilla F, Schneider L (2005). TACI is mutant in common variable immunodeficiency and IgA deficiency. Nat Genet.

[51] Grimbacher B, Hutloff A, Schlesier M, Glocker E, Warnatz K, Drager R (2003). Homozygous loss of ICOS is associated with adult-onset common variable immunodeficiency. Nat Immunol.

[52] Warnatz K, Denz A, Drager R, Braun M, Groth C, Wolff-Vorbeck G (2002). Severe deficiency of switched memory B cells (CD27(+)IgM(-)IgD(-)) in subgroups of patients with common variable immunodeficiency: A new approach to classify a heterogeneous disease. Blood.

[53] Cunningham-Rundles C, Bodian C (1999). Common variable immunodeficiency: Clinical and immunological features of 248 patients. Clin Immunol.

[54] Lai Ping So A, Mayer L (1997). Gastrointestinal manifestations of primary immunodeficiency disorders. Semin Gastrointest Dis.

[55] Daniels JA, Lederman HM, Maitra A, Montgomery EA (2007). Gastrointestinal tract pathology in patients with common variable immunodeficiency (CVID): A clinicopathologic study and review. Am J Surg Pathol.

[56] Zullo A, Romiti A, Rinaldi V, Vecchione A, Tomao S, Aiuti F (1999). Gastric pathology in patients with common variable immunodeficiency. Gut.

[57] Washington K, Stenzel TT, Buckley RH, Gottfried MR (1996). Gastrointestinal pathology in patients with common variable immunodeficiency and X-linked agammaglobulinemia. Am J Surg Pathol.

[58] Kinlen LJ, Webster AD, Bird AG, Haile R, Peto J, Soothill JF (1985). Prospective study of cancer in patients with hypogammaglobulinaemia. Lancet.

[59] Chiaramonte C, Glick SN (1994). Nodular lymphoid hyperplasia of the small bowel complicated by jejunal lymphoma in a patient with common variable immune deficiency syndrome. AJR Am J Roentgenol.

[60] Erdos M, Durandy A, Marodi L (2005). Genetically acquired class-switch recombination defects: The multi-faced hyper-IgM syndrome. Immunol Lett.

[61] Allen RC, Armitage RJ, Conley ME, Rosenblatt H, Jenkins NA, Copeland NG (1993). CD40 ligand gene defects responsible for X-linked hyper-IgM syndrome. Science.

[62] DiSanto JP, Bonnefoy JY, Gauchat JF, Fischer A, de Saint Basile G (1993). CD40 ligand mutations in x-linked immunodeficiency with hyper-IgM. Nature.

[63] Korthauer U, Graf D, Mages HW, Briere F, Padayachee M, Malcolm S (1993). Defective expression of T-cell CD40 ligand causes X-linked immunodeficiency with hyper-IgM. Nature.

[64] Ferrari S, Giliani S, Insalaco A, Al-Ghonaium A, Soresina AR, Loubser M (2001). Mutations of CD40 gene cause an autosomal recessive form of immunodeficiency with hyper IgM. Proc Natl Acad Sci USA.

[65] Jain A, Ma CA, Liu S, Brown M, Cohen J, Strober W (2001). Specific missense mutations in NEMO result in hyper-IgM syndrome with hypohydrotic ectodermal dysplasia. Nat Immunol.

[66] Revy P, Muto T, Levy Y, Geissmann F, Plebani A, Sanal O (2000). Activation-induced cytidine deaminase (AID) deficiency causes the autosomal recessive form of the Hyper-IgM syndrome (HIGM2). Cell.

[67] Imai K, Slupphaug G, Lee WI, Revy P, Nonoyama S, Catalan N (2003). Human uracil-DNA glycosylase deficiency associated with profoundly impaired immunoglobulin class-switch recombination. Nat Immunol.

[68] Levy J, Espanol-Boren T, Thomas C, Fischer A, Tovo P, Bordigoni P (1997). Clinical spectrum of X-linked hyper-IgM syndrome. J Pediatr.

[69] Quartier P, Bustamante J, Sanal O, Plebani A, Debre M, Deville A (2004). Clinical, immunologic and genetic analysis of 29 patients with autosomal recessive hyper-IgM syndrome due to Activation-Induced Cytidine Deaminase deficiency. Clin Immunol.

[70] Debray D, Pariente D, Urvoas E, Hadchouel M, Bernard O (1994). Sclerosing cholangitis in children. J Pediatr.

[71] Hayward AR, Levy J, Facchetti F, Notarangelo L, Ochs HD, Etzioni A (1997). Cholangiopathy and tumors of the pancreas, liver, and biliary tree in boys with X-linked immunodeficiency with hyper-IgM. J Immunol.

[72] Wildin RS, Ramsdell F, Peake J, Faravelli F, Casanova JL, Buist N (2001). X-linked neonatal diabetes mellitus, enteropathy and endocrinopathy syndrome is the human equivalent of mouse scurf y. Nat Genet.

[73] Bennett CL, Christie J, Ramsdell F, Brunkow ME, Ferguson PJ, Whitesell L (2001). The immune dysregulation, polyendocrinopathy, enteropathy, X-linked syndrome (IPEX) is caused by mutations of FOXP3. Nat Genet.

[74] Myers AK, Perroni L, Costigan C, Reardon W (2006). Clinical and molecular findings in IPEX syndrome. Arch Dis Child.

[75] Gambineri E, Torgerson TR, Ochs HD (2003). Immune dysregulation, polyendocrinopathy, enteropathy, and X-linked inheritance (IPEX), a syndrome of systemic autoimmunity caused by mutations of FOXP3, a critical regulator of T-cell homeostasis. Curr Opin Rheumatol.

[76] Wildin RS, Smyk-Pearson S, Filipovich AH (2002). Clinical and molecular features of the immunodysregulation, polyendocrinopathy, enteropathy, X linked (IPEX) syndrome. J Med Genet.

[77] Bennett CL, Ochs HD (2001). IPEX is a unique X-linked syndrome characterized by immune dysfunction, polyendocrinopathy, enteropathy, and a variety of autoimmune phenomena. Curr Opin Pediatr.

[78] Baud O, Goulet O, Canioni D, Le Deist F, Radford I, Rieu D (2001). Treatment of the immune dysregulation, polyendocrinopathy, enteropathy, X-linked syndrome (IPEX) by allogeneic bone marrow transplantation. N Engl J Med.

[79] Casanova JL, Abel L (2009). Revisiting Crohn's disease as a primary immunodeficiency of macrophages. J Exp Med.

[80] Marks DJ, Rahman FZ, Sewell GW, Segal AW (2010). Crohn's disease: an immune deficiency state. Clin Rev Allergy Immunol.

[81] Smith AM, Rahman FZ, Hayee B, Graham SJ, Marks DJ, Sewell GW (2009). Disordered macrophage cytokine secretion underlies impaired acute inflammation and bacterial clearance in Crohn's disease. J Exp Med.

[82] Hugot JP, Chamaillard M, Zouali H, Lesage S, Cezard JP, Belaiche J (2001). Association of NOD2 leucine-rich repeat variants with susceptibility to Crohn's disease. Nature.

[83] Ogura Y, Bonen DK, Inohara N, Nicolae DL, Chen FF, Ramos R (2001). A frameshift mutation in NOD2 associated with susceptibility to Crohn's disease. Nature.

